# Urbanization and kidney function decline in low and middle income countries

**DOI:** 10.1186/s12882-017-0685-4

**Published:** 2017-08-29

**Authors:** Ram Jagannathan, Rachel E. Patzer

**Affiliations:** 1 Global Diabetes Research Center, School of Public Health, Rollins School of Public Health, Atlanta, GA USA; 2 Department of Surgery, Division of Transplantation, Emory University School of Medicine, 101 Woodruff Circle, 5101 Woodruff Memorial Research Building, Atlanta, GA USA

**Keywords:** Chronic kidney disease, Low-Middle income countries, Urbanization, Screening, Management

## Abstract

Urbanization is expected to increase in low and middle-income countries (LMICs), and might contribute to the increased disease burden. The association between urbanization and CKD is incompletely understood among LMICs. Recently, Inoue et al.*,* explored the association of urbanization on renal function from the China Health and Nutrition Survey. The study found that individuals living in an urban environment had a higher odds of reduced renal function independent of behavioral and cardiometabolic measures, and this effect increased in a dose dependent manner. In this commentary, we discuss the results of these findings and explain the need for more surveillance studies among LMICs.

## Background

*The cities of the developing world are spectacularly ill-prepared for the explosion in urban living* – Hans van Ginkel [1]. For the first time in human history, more than half of the world’s population resides in urban areas, a proportion that is expected to increase to 66% in the next three decades [2]. The United Nations estimated that the urban population will further increase from 3.3 to 4.9 billion worldwide by 2030 [2]. Various studies in low and middle income countries (LMICs) have found that rapid and unplanned urbanization may lead to higher prevalence of non-communicable diseases such as obesity, diabetes, hypertension and dyslipidemia [3, 4]. This upsurge in metabolic diseases parallels the rise in prevalence of chronic kidney disease (CKD). According to the available evidence, approximately 500 million people are estimated to have CKD, with the majority (>80%) living in LMICs, but these estimates belie the true burden of the disease [5]. Correspondingly, the age-standardized mortality rate owing to CKD increased by 37% over two decades (Year:1990: 11.6 deaths per 100,000 population; Year: 2013: 15.8 deaths per 100,000 population), making CKD the 19th leading global cause of years of life lost in 2013 [6]. However, the data on CKD epidemiology remains incompletely understood, due to availability of few surveillance data, poor standardization of creatinine and albuminuria measurements, and inconsistent methods for ascertaining kidney function. Based on the available evidence from LMICs, there is substantial heterogeneity in the prevalence of CKD among urban versus rural areas (Table 1).

**Table 1:** Prevalence of chronic kidney disease among low and middle income countries stratified by urban-rural geography

Country, year [ref]	Ethnicity	Criteria/classification used	Overall (%)	Urban (%)	Rural (%)
Cameroon, 2013 [24]	African	any albuminuria and/or eGFR < 60 ml/min	14.2	14.2	NA
Cameroon, 2014 [25]	African	MDRD	13.2	10.9	14.1
Tanzania, 2014 [26]	African	MDRD/albuminuria	7.0	15.2	2.0
Sub-Saharan systematic review, 2014	Sub-Saharan	MDRD/CKD-EPI and albuminuria	13.9	12.4	16.5
Thailand, 2004 [27]	Asian	MDRD	8.5	8.0	9.2
Thailand, 2008 [28]	Asian	Spot quantitative urine protein and eGFR (MDRD)	17.5	23.1	15.8
Thailand, 2006 [29]	Asian	eGFR (MDRD)	13.2		
Turkey, 2005 [30]	Turks	eGFR (MDRD)	5.8	4.9	6.6
India, 2005 [31]	Asian	eGFR (MDRD)	17.2		
India, 2016 [32]	Asian	CG-BSA	16.5	NA	16.54
China, 2001 [33]	Asian	eGFR (MDRD)	2.53	2.60	2.52
China, 2006 [34]	Asian	eGFR (MDRD)/albuminuria	12.1	12.1	
China, 2006 [35]	Asian	eGFR (MDRD)/albuminuria	15.2		15.2
China, 2006 [36]	Asian	eGFR (MDRD) and albuminuria	13.0	13.0	13.1
China, 2009 [37]	Asian	eGFR (MDRD)/albuminuria	10.8	8.9	11.3
China, 2010 [38]	Asian	eGFR (MDRD)/albuminuria	9.50	9.58	9.42

## Main text

Unlike western countries, the etiology of CKD in LMICs are multifactorial and affected by the double burden of both non-communicable and communicable diseases. Table 2 describes some of the etiological factors associated with CKD among LMICs. Adverse lifestyle changes, including increased sedentary habits [7], lack of sleep [8], dietary sodas [9], high intake of calorie and sodium-rich diets [10, 11], food insecurity and poverty [12], and increased availability of processed foods [13] results in increased prevalence of non-communicable diseases. Accordingly, about half of all deaths in Asia are now attributable to non-communicable diseases, accounting for 47% of global burden [14]. Conversely, a preponderance of infectious diseases not typically seen in high income countries, such as schistosomiasis, HIV, tuberculosis, hepatitis B and C, further aggravates the CKD burden in LMICs [15]. Furthermore, rapid and unplanned urbanization may result in decreased food availability for socially disadvantaged people, lack of infrastructure, poor sanitation, waste disposal and heavy environmental toxins [16]. Inadequate health care resources, coupled with increased prevalence of other communicable and NCDs, further exacerbate the burden [17]. Genetic predisposition [18], malnutrition [19] and intrauterine fetal exposures [20] also play an important role in CKD susceptibility.

**Table 2:** Known potential causes of CKD in LMICs

Risk factors
I. Lifestyle a. Sedentary lifestyle b. Increased caloric intake c. High intake of calorie-rich foods, sodas, red meat and decreased intake of fruits and vegetables d. High sodium intake e. Lack of sleep
II. Non-Communicable Diseases a. Obesity b. Diabetes, Type II c. Hypertension d. Poor management of diabetes and hypertension (poor medication adherence)
III. Environmental Factors a. Food insecurity and poverty b. Air/water pollution and industrial waste products c. Heavy metals (lead, Cadmium, arsenic, gold, mercury and uranium) d. Plastics and resins (Bisphenols) e. Over-the-counter drug and Counterfeit drugs f. Pesticides, hardness of water, g. Superphosphates, h. Arsenic-contaminated fertilizers and cyanogens from algae
IV. Infections a. Tuberculosis b. Hepatitis B and C c. Malaria and other viral vector borne diseases (dengue and yellow fever) d. Parasitic diseases including schistosomiasis, filariasis, and leishmaniasis

In this issue of the journal, Inoue et al. [21]*,* studied the effect of urbanization on renal function from the China Health and Nutrition Survey (*n* = 9493). Rather than relying on a conventional urban-rural dichotomous measure, the authors utilized a validated, study specific, 12-component composite index to measure urbanization. The study showed that individuals living in an urban environment had higher odds of reduced glomerular filtration rate, independent of behavioral and cardiometabolic measures. Of particular note, the effect of urbanization on renal function decline was dose-dependent, where mean eGFR (ml/min/1.73m^2^) declined from 84.8 in the lowest urbanization quartile to 82.8, 80.9, and then 77.8 in the highest urbanization quartile. In multivariable, multilevel models, the impact of a 1 standard deviation increase in urbanization was associated with reduced renal function among both males and females (males: adjusted odds ratio (aOR): 1.25 [95% CI: 0.98–1.59]; *P* = 0.078; females: aOR: 1.24 [95% CI: 1.01–1.52]; *P* = 0.041). Despite lack of statistical significance among males, the effect sizes were similar. In addition, among the different components of the urbanization index, the authors found a significant association between housing component and renal function (males: OR: 1.51 [95% CI: 1.01–2.28] and females: OR: 1.39 [95% CI: 1.01–1.93]). While the mechanism for how increased urbanization can lead to lower renal function is not possible to explore in this cross-sectional study, results suggest that community-level factors may play an important role in the development of CKD and should be further explored.

Addressing NCDs is an urgent global priority, and cities are the epicenters where action is needed most. Compared with rural areas, the rapid urbanization and lifestyle changes in cities offers better understanding of the etiology of the CKD diseases. Despite a probable heavy burden of end-stage kidney disease in LMICs, relatively few patients receive renal replacement therapy. A 2015 systematic review suggests that approximately 83% of renal failure patients in Asia are not receiving the renal replacement treatment [22]. A recent Cochrane review which included 17 studies involving 1639 people with CKD showed that different modalities of dietary interventions were associated with improved kidney function and hemodynamic measures [23]. However, none of the studies were reported from LMICs. Innovation, technology, and the ongoing economic transition in cities may also help to catalyze new initiatives to prevent CKD burden in this countries. Future studies should focus on the following themes:

- Better surveillance studies are urgently needed to ascertain the real burden of CKD prevalence among LMICs
- Because the presentation of CKD is heterogeneous among LMICs, a better understanding of the epidemiology is urgently needed. Non-conventional risk factors such as heavy metals, environmental toxins, sleep insufficiency and stress should be explored as factors mediating urbanization and CKD; and
- The available CKD nutritional intervention studies are posited based on evidence from homogoenous populations in higher income countries and therefore, more studies are needed to test the effectiveness of lifestyle interventions among LMICs. The studies should particularly focused on testing culturally tailored and novel dietary intervention tools.

## Conclusions

To conclude, CKD is an important global health challenge especially in LMICs. National and international efforts are urgently needed on the prevention, detection and treatment to mitigate the rising burden worldwide. Furthermore, a better understanding of epidemiology coupled with strong public advocacy, and collaborative public health interventions that address conventional and unconventional risk factors of CKD are needed. Future studies should focus on the unique features of LMICs to ascertain those at greatest risk of CKD, across all socioeconomic groups to ensure early screening.

## Abbreviations

CKD: Chronic kidney disease
eGFR: Estimated glomerular filtration rate
LMIC: Low-Middle income countries
NCD: Non-communicable disease
